# Intracardiac paraganglioma with a cough as the first symptom

**DOI:** 10.1186/s13019-022-02087-z

**Published:** 2023-01-17

**Authors:** Cao Jingyi, Xue Qing, Yang Fan, Yang Qinqin, Cai Chengliang, Lu Fanglin

**Affiliations:** 1 Department of Cardiovascular Surgery, Changhai Hospital, Naval Medical University, Shanghai, 200433 People’s Republic of China; 2 Department of Nuclear Medicine, Changhai Hospital, Naval Medical University, Shanghai, 200433 People’s Republic of China

**Keywords:** Cardiac neoplasm, Paraganglioma, Cardiovascular surgical procedure, Catecholamine, Gene mutation

## Abstract

**Background:**

Cardiac paragangliomas (PGLs) are clinically rare, with hypertension and metabolic changes as the main symptoms. The tumor is highly related to gene mutation, and surgery is presently the effective treatment. Medical history and clinical manifestations of the patient, routine laboratory examinations and imaging examinations, and pathological examination can help the final diagnosis.

**Case presentation:**

The present study presents a 31-year-old male patient with a left atrial mass. The initial symptom was cough. Cardiac enlargement was found during the chest X-ray. The follow-up imaging examination revealed a left atrial occupying lesion, and the possibility of malignant occupying lesions was not ruled out. The patient underwent surgical resection of the mass. The final pathological result revealed paraganglioma. The thoracic computed tomography review two months after the operation revealed that the original occupying lesion disappeared, and no new lesion was found.

**Conclusions:**

Pheochromocytomas and paragangliomas (PPGLs) are a kind of neuroendocrine tumors. PPGLs can cause secondary hypertension, and lead to a series of clinical syndromes, including myocardial injury, metabolic changes, and so on. The occurrence of PPGIs is related to gene mutation. Biochemical detection, imaging examination, and genetic testing can help diagnose. The tumor should be surgically removed as soon as possible after the diagnosis. As a functional tumor, PPGLs should be fully prepared before surgery to avoid anesthesia and huge fluctuations in blood pressure during and after surgery, or the occurrence of fatal hypertensive crisis and intractable hypotension after tumor resection. Adequate preoperative preparation directly affects the prognosis of patients after surgery. Therefore, multidisciplinary cooperation before, during, and after the operation is extremely important.

## Background

Pheochromocytomas and paragangliomas (PPGLs) are a class of neuroendocrine tumors that originate in the adrenal medulla and extra-adrenal sympathetic chain, respectively. PPGLs are capable of the paroxysmal or persistent secretion of catecholamines, causing secondary hypertension and contributing to a range of clinical syndromes, including myocardial damage, metabolic alterations, and so on [1]. PPGLs are uncommon, with an incidence of about 8 cases per million people [2]. It has been convinced that PPGLs are the most heritable cancer type, with 40% of patients carrying predisposing mutations [3]. Surgery is presently the effective treatment of choice for PPGLs [4]. However, owing to its rarity, there have been few appropriate guidelines for the evaluation, preparation, and surgical approach to these tumors. The present report describes a case of intracardiac paraganglioma, cured by surgery, with a cough as the initial symptom.

## Case presentation

A 31-year-old male was referred to our hospital with a tumor in the left atrium (LA). Half a month before coming to our hospital, the patient went to a local hospital for treatment due to an intermittent cough. The chest X-ray revealed no obvious inflammation in the lungs, but the heart was dilated. The transthoracic echocardiography indicated a left atrial occupying lesion and a large amount of pericardial effusion. Pericardiocentesis was performed, and 600 mL of bloody pericardial effusion was removed. Then, the patient visited our hospital for further treatment.

**Fig. 1:**
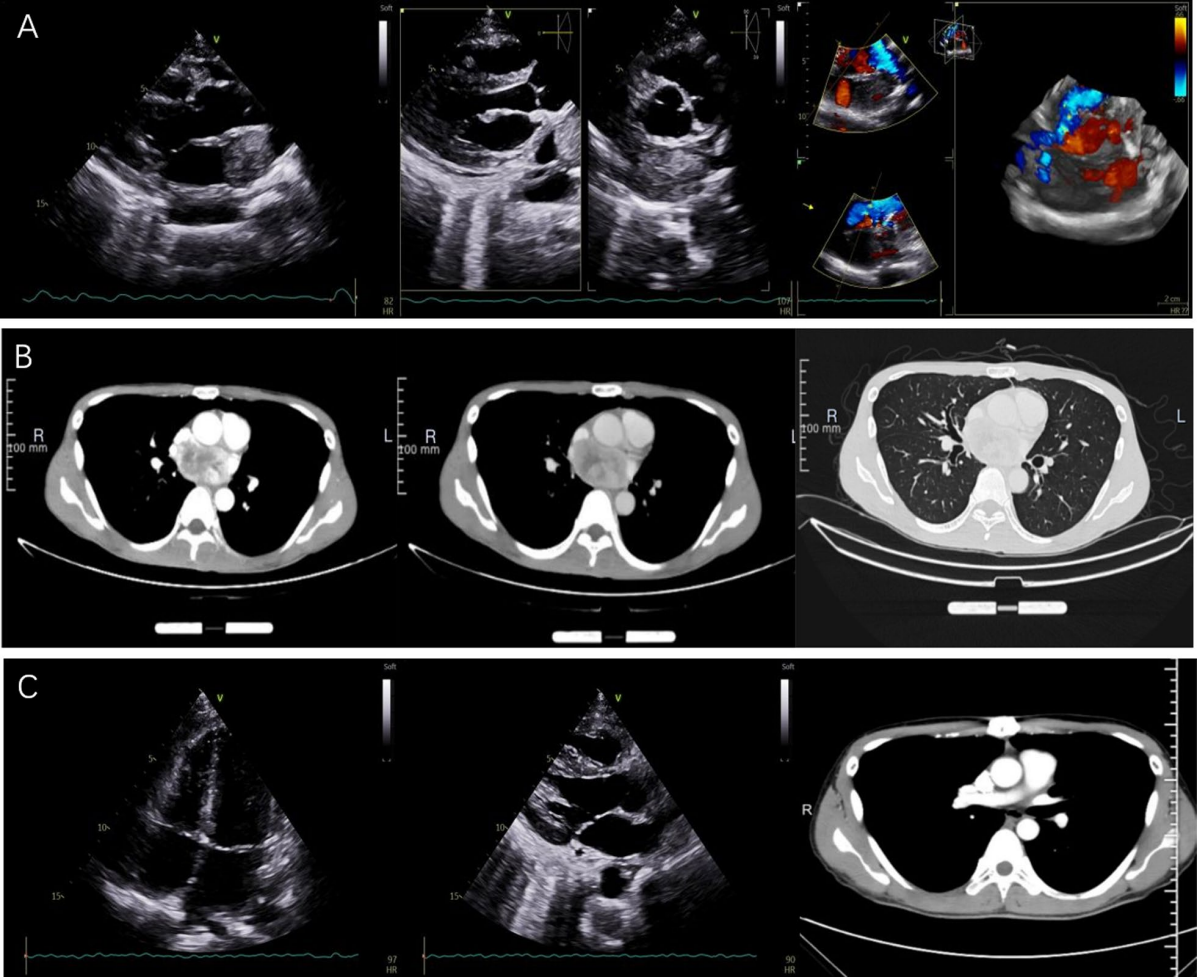
Preoperative imaging of the mass at transthoracic echocardiography (**A**) and CT (**B**). Postoperative transthoracic echocardiography and CT imaging (**C**)

**Fig. 2:**
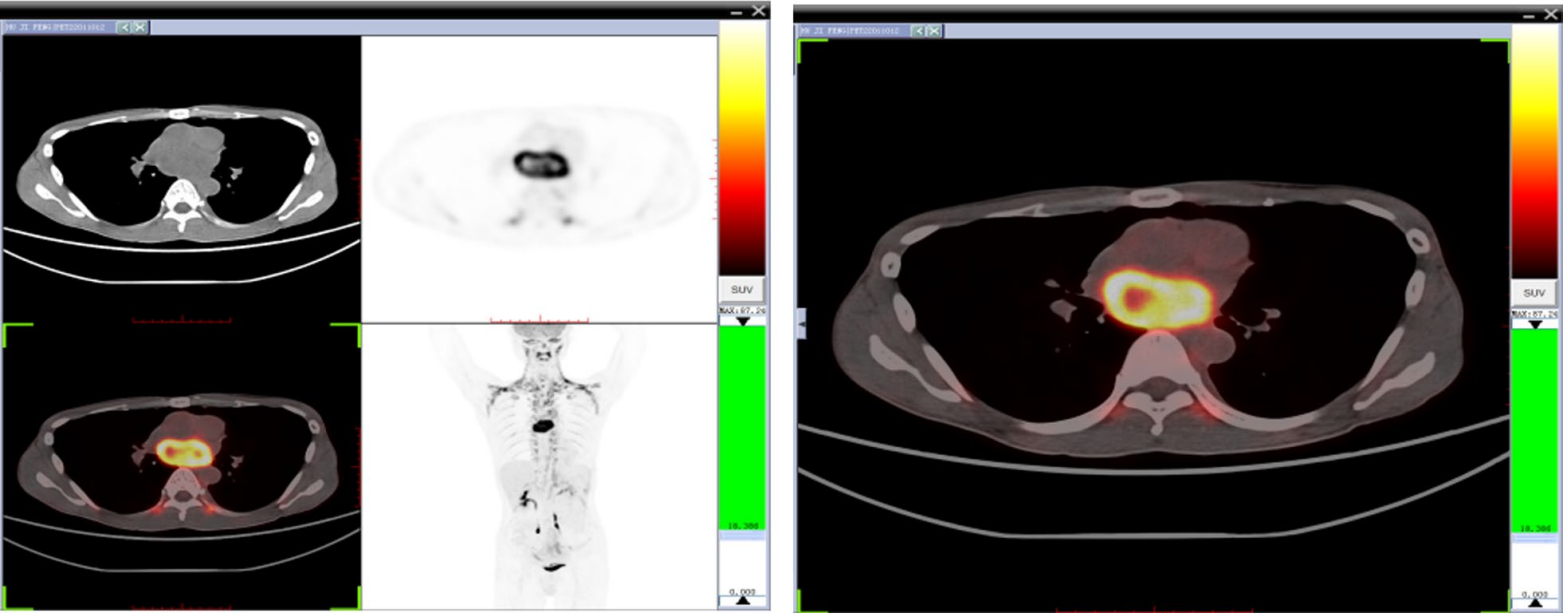
PET-CT The arrow indicates a hypermetabolic mass (3.5 * 6.5 cm) in the left atrium

The patient had no family history related to tumors or environmental exposure histories, such as chemical substances and high radiation. The patient lost 5 kg of weight since its onset. Routine laboratory examinations were performed after admission (Table 1). The transthoracic echocardiography (Fig. 1A) revealed an occupying lesion (2.8 × 4.5 cm) in the LA, with an estimated ejection fraction (EF) of 80%. Pleural effusion was detected in the left chest, with a depth of 5 cm, and 100 mL of bloody effusion was obtained by thoracocentesis. To determine the boundary between the occupying lesion and the heart, thoracic computed tomography (CT) was employed. This revealed the left atrial occupying lesion (3.44 × 5.54 cm), and the inhomogeneous enhancement became visible after the image enhancement (Fig. 1B). At the same time, a strange phenomenon was observed: when the blood pressure was monitored by routine nursing, the systolic blood pressure of the patient reached as high as 180–200 mmHg. This phenomenon often occurred when the blood pressure was measured after an activity. The blood pressure returned to normal levels after several minutes of rest. The investigators highly suspected that this was a catecholamine-related neoplasm. We chose α-blockers for antihypertensive therapy. Catecholamine-related testing was not set up in our hospital. Furthermore, due to the COVID-19 outbreak, the patient could not go to other hospitals for examination. Therefore, the investigators decided to perform positron emission tomography (PET)-CT to clarify the nature of the tumor, and assist in the diagnosis. The final results suggested a soft tissue mass (3.5 × 6.5 cm) in the LA, and a malignant occupying lesion was possible. In addition, it was reported that there were hypermetabolic regions of brown adipose tissues in the neck, shoulder and thoracolumbar paravertebral areas (Fig. 2). The PET-CT results further suggested the possibility of a catecholamine-related tumor.

**Table 1:** Routine laboratory examinations

	Preoperative	Postoperative
White blood cell (× 10^9^/L)	12.01	10.93
Red blood cell (× 10^12^/L)	4.98	3.32
Platelet (× 10^9^/L)	415	129
Albumin (g/L)	39	34
Globulin (g/L)	35	26
International normalized ratio (INR)	1.11	1.14
Brain natriuretic peptide (BNP) (pg/mL)	69.34	128.22

**Fig. 3:**
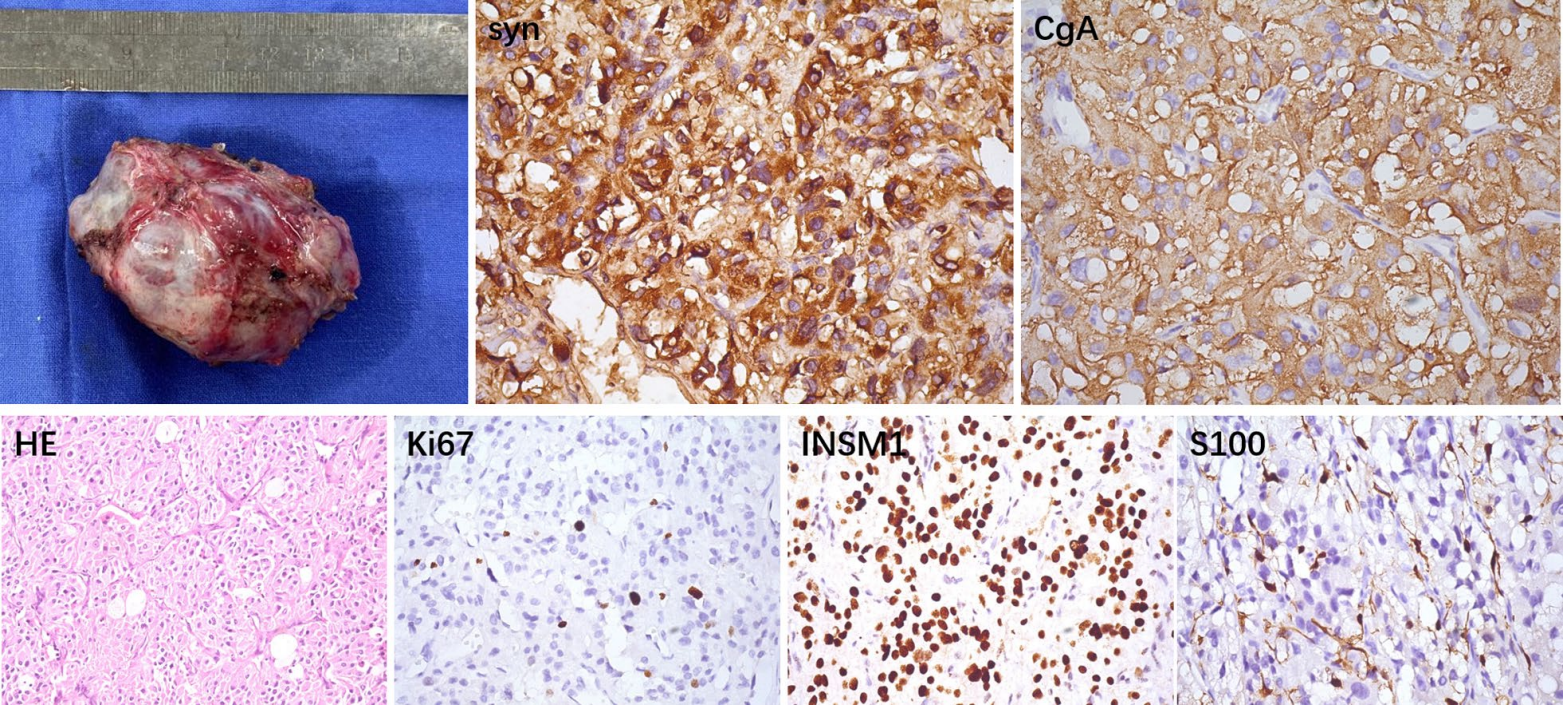
The tumor measured approximately 6.5 cm in length. The histological examination and immunohistochemical analysis (400× magnification) showed a final result of a paraganglioma

After a series of examinations, the investigators decided to surgically remove the tumor. During the preoperative anesthesia, the blood pressure remained at a high level, despite the drug intervention. After the general anesthesia, the investigators performed a median incision on the chest and exposed the heart position. After establishing the cardiopulmonary bypass, the right atrium and atrial septum were incised, and it could be observed that the intima of the LA was smooth and intact. Then, the location of the tumor was explored. It was observed that the tumor was located within the wall of the LA, and adhered to the right pulmonary artery. The tumor was carefully separated from the heart adhesion to completely remove the tumor. Finally, left atrial reconstruction was performed with commoditized bovine pericardium. It is noteworthy that during the surgery, regardless of whenever the tumor was touched, the patient’s blood pressure transiently raised. The tumor was approximately 6.5 cm in length. The removed tumor was submitted for pathological analysis, and the final result was suggestive of paraganglioma (Fig. 3). The patient’s condition was stable after the surgery, and no other special treatment drugs were used. The postoperative echocardiogram suggested (Fig. 1C) that after the left atrial tumor resection and left atrial reconstruction, each atrium and ventricle of the heart returned to the normal size. The thoracic CT review revealed that the original occupying lesion disappeared, and no new occupying lesions were detected (Fig. 1C). The results of the catecholamine concentration tested in other hospitals after the operation were, as follows: dopamine, < 0.16 nmol/L; epinephrine, 0.35 nmol/L; norepinephrine, 0.92 nmol/L. These were within the normal range.

## Discussion and conclusions

Pheochromocytomas (PCCs) are located in the adrenal glands, while paragangliomas (PGLs) are located along the paraspinal sympathetic chain and are classified as hormonally active or inactive, and both of which are jointly known as PPGLs [1, 5]. The annual incidence of PPGLs is approximately eight per million, with a rate of 2% happening in the chest [6, 7]. Most paragangliomas found in the chest occur in the posterior mediastinum [8, 9]. In general, cardiac paragangliomas are extremely rare, accounting for less than 1% of all cardiac tumors [10]. PPGLs are considered to occur in 0.05–0.10% of patients with persistent hypertension. However, this accounts for merely 50% of patients with PPGL, since the rest only had paroxysmal hypertension or normal blood pressure [1]. The disease is usually diagnosed between the age of 30 and 50, and approximately 10–49% of patients are incidentally discovered during imaging studies for other reasons [11, 12].

Although rare, PPGLs are the most heritable cancer type, with 40% of patients carrying predisposing germline mutations [3]. In 2017, the World Health Organization (WHO) replaced the “malignant PPGLs” defined in 2004 with “metastatic PPGLs” in the classification of neuroendocrine tumors [13]. However, the mechanisms of metastasis related to genes have not been clearly defined. At least 17 different genes have shown some relationships with PGLs [5]. Succinate dehydrogenase (SDH) has been proven to be correlated with cardiac PGLs, especially SDHB and SDHD which are highly susceptible genes in left atrium PGLs. These findings may be useful in making a follow-up plan and achieving proper treatment.

The main signs and symptoms of excess circulating catecholamines from PPGL are headache, palpitations, sweating, pallor, nausea, constipation, flushing, weight loss, fatigue, anxiety, sustained or paroxysmal hypertension, orthostatic hypotension, fever, and hyperglycemia [1, 14]. These episodes are correlated to the excess catecholamine produced by the tumor. An unexplained orthostatic hypotension on the background of paroxysmal or refractory hypertension may be an important diagnostic clue that may help clinicians to suspect this tumor [15]. The present report describes a rare case of intracardiac paraganglioma. The patient was hospitalized with a cough, which is a rare symptom of a catecholamine-associated tumor that may have been caused by the irritation of the trachea induced by the pericardial effusion that resulted from the tumor. The occasional transient rise in blood pressure during the diagnosis and treatment suggests the possibility of a catecholamine-related tumor, and this was confirmed by the PET-CT. In addition, to our knowledge, brown adipose intake is diverse, and mostly occurs in cold seasons and young, female subjects with low body mass index (BMI). When the abnormal catecholamine-related secretion is present, brown adipose intake can also occur, which often symmetrically involves the neck and shoulder, supraclavicular area, both sides of the spine, the surrounding mediastinal great vessels, the adrenal area, and the perirenal area [16]. Therefore, after excluding relevant factors, such as gender and environment, the investigators had more reason to consider that this was a catecholamine-related tumor. Regrettably, due to the COVID-19 outbreak, the patient was unable to carry out the preoperative catecholamine concentration test in other hospitals.

The present case provides valuable experience for the treatment of similar patients in the future. After the diagnosis, the tumor should be removed by surgery as early as possible. Surgery can improve the symptoms, reduce hormone secretion, prevent associated complications, and improve the outcome of subsequent treatments. However, crushing the tumor during surgery can lead to the massive release of catecholamines, which may cause a hypertensive crisis, arrhythmia, stroke, etc. Furthermore, postoperatively, the resulting dramatic reduction in catecholamines may cause peripheral vasodilatation, causing persistent hypotension, and even death. Therefore, adrenergic α-blockers can be routinely used preoperatively, and β-blockers can be added if the patient develops tachycardia [17]. The use of β-blockers alone is not allowed to prevent acute cardiac insufficiency. Adrenergic α-blockers can be used when hypertension occurs during surgery, and β-blockers can be added if arrhythmia occurs [18]. If hypotension occurs after tumor resection, α-blockers should be discontinued immediately and blood volume replenishment should be performed. Blood pressure and heart rate should be closely monitored after surgery, and hypertension can be cured in most patients.

As a consequence, during the patient’s hospitalization, multidisciplinary consultation should be strengthened, including the Endocrine Department, Intensive Medicine and Anesthesiology Department, and other disciplines, in order to ensure patient safety maximization. Furthermore, postoperative long-term follow-ups should be conducted.

## Data Availability

The datasets used and/or analyzed during the current study are available from the corresponding author on reasonable request.

## Abbreviations

PPGLs: Pheochromocytomas and paragangliomas
LA: Left atrium
INR: International normalized ratio
EF: Ejection fraction
CT: Computed tomography
PET: Positron emission tomography
PCCs: Pheochromocytomas
PGLs: Paragangliomas
SDH: Succinate dehydrogenase

## References

[1] Chen H, Sippel RS, O’Dorisio MS (2010). The North American neuroendocrine tumor society consensus guideline for the diagnosis and management of neuroendocrine tumors: pheochromocytoma, paraganglioma, and medullary thyroid cancer. Pancreas.

[2] Aravot DJ, Banner NR, Cantor AM, Theodoropoulos S, Yacoub MH (1992). Location, localization and surgical treatment of cardiac pheochromocytoma. Am J Cardiol.

[3] Crona J, Taïeb D, Pacak K (2017). New perspectives on pheochromocytoma and paraganglioma: toward a molecular classification. Endocr Rev.

[4] Wang JG, Han J, Jiang T, Li YJ (2015). Cardiac paragangliomas. J Card Surg.

[5] Chan EY, Ali A, Umana JP (2022). Management of primary cardiac paraganglioma. J Thorac Cardiovasc Surg.

[6] Brown ML, Zayas GE, Abel MD, Young WF, Schaff HV (2008). Mediastinal paragangliomas: the mayo clinic experience. Ann Thorac Surg.

[7] Walther MM, Keiser HR, Linehan WM (1999). Pheochromocytoma: evaluation, diagnosis, and treatment. World J Urol.

[8] Manger WM, Gifford RW (1982). Hypertension secondary to pheochromocytoma. Bull N Y Acad Med.

[9] Hamilton BH, Francis IR, Gross BH (1997). Intrapericardial paragangliomas (pheochromocytomas): imaging features. AJR Am J Roentgenol.

[10] Khan MF, Datta S, Chisti MM, Movahed MR (2013). Cardiac paraganglioma: clinical presentation, diagnostic approach and factors affecting short and long-term outcomes. Int J Cardiol.

[11] Baguet JP, Hammer L, Mazzuco TL (2004). Circumstances of discovery of phaeochromocytoma: a retrospective study of 41 consecutive patients. Eur J Endocrinol.

[12] Kopetschke R, Slisko M, Kilisli A (2009). Frequent incidental discovery of phaeochromocytoma: data from a German cohort of 201 phaeochromocytoma. Eur J Endocrinol.

[13] Lam AKY (2017). Update on adrenal tumours in 2017 World Health Organization (WHO) of endocrine tumours. Endocr Pathol.

[14] Lenders JWM, Eisenhofer G, Mannelli M, Pacak K (2005). Phaeochromocytoma. Lancet.

[15] Desai AS, Chutkow WA, Edelman E, Economy KE, Dec GW (2009). Clinical problem-solving. A crisis in late pregnancy. N Engl J Med.

[16] Dong A, Wang Y, Lu J, Zuo C (2014). Hypermetabolic mesenteric brown adipose tissue on dual-time point FDG PET/CT in a patient with benign retroperitoneal pheochromocytoma. Clin Nucl Med.

[17] Lenders JWM, Duh QY, Eisenhofer G (2014). Pheochromocytoma and paraganglioma: an endocrine society clinical practice guideline. J Clin Endocrinol Metab.

[18] Naranjo J, Dodd S, Martin YN (2017). Perioperative Management of Pheochromocytoma. J Cardiothorac Vasc Anesth.

